# Therapeutic effects and underlying mechanism of poly (L-glutamic acid)-*g*-methoxy poly (ethylene glycol)/combretastatin A4/BLZ945 nanoparticles on Renca renal carcinoma

**DOI:** 10.3389/fbioe.2024.1336692

**Published:** 2024-02-05

**Authors:** Jiaqi Chen, Min Yin, Chenguang Yang, Kun Wang, Lili Ma, Haiyang Yu, Yue Huang, Feng Liu, Zhaohui Tang

**Affiliations:** ^1^ Department of Nephrology, China-Japan Union Hospital of Jilin University, Jilin University, Changchun, China; ^2^ Key Laboratory of Polymer Ecomaterials, Changchun Institute of Applied Chemistry, Chinese Academy of Sciences, Changchun, China

**Keywords:** combretastatin A4, BLZ945, Renca renal carcinoma, therapeutic effect, mechanism

## Abstract

**Introduction:** The prognosis of advanced renal carcinoma is not ideal, necessitating the exploration of novel treatment strategies. Poly(L-glutamic acid)-g-methoxy poly(ethylene glycol)/Combretastatin A4 (CA4)/BLZ945 nanoparticles (CB-NPs) possess the dual capability of CA4 (targeting blood vessels to induce tumor necrosis) and BLZ945 (inducing M2 macrophage apoptosis), thereby inhibiting tumor growth.

**Methods:** Here, the therapeutic effects and underlying mechanism was explored by CCK-8 cytotoxicity experiment, transwell cell invasion and migration experiment, H&E, western blot analysis, immunohistochemistry, flow cytometry, and other techniques.

**Results:** These results demonstrated that CB-NPs could inhibit the growth of Renca cells and subcutaneous tumors in mice, with an impressive tumor inhibition rate of 88.0%. Results suggested that CB-NPs can induce necrosis in renal carcinoma cells and tissues, downregulate VEGFA expression, promote renal carcinoma cell apoptosis, and reduce the polarization of M2 macrophages.

**Discussion:** These findings offer innovative perspectives for the treatment of advanced renal carcinoma.

## 1 Introduction

Renal carcinoma is a prevalent malignant tumor within the urinary system. Its global incidence rate has been on the rise (Capitanio et al., 2019). Early-stage renal cell carcinoma presents as asymptomatic, resulting in diagnosis at advanced stages. Surgical treatment is the preferred option for managing renal carcinoma, while immunotherapies have shown sensitivity instead of chemotherapy drugs (Klapper et al., 2008; Aggen et al., 2021). Currently, the prognosis of targeted drugs that combined to inhibit angiogenesis is unsatisfactory (Maslov et al., 2022; Li et al., 2023). Previous studies have shown that renal carcinoma is a malignant tumor that relies on angiogenesis and hypoxia. As a result, certain molecular targeted drugs are used to disrupt crucial pathways in renal carcinoma development, such as multi-receptor tyrosine kinase (RTK) inhibitors and mTOR pathway inhibitors (Baldewijns et al., 2010). However, drug resistance often develops in advanced patients, leading to treatment failure and the development of refractory diseases (Wilson and Hay, 2011; Ellis et al., 2012). Therefore, developing novel treatment strategies remains the focus of the diagnosis and treatment of renal carcinoma.

CA4 nanomedicines (C-NPs) represent prodrugs of vascular disrupting agents (VDAs) that primarily localize CA4 around blood vessels with certain degrees of tumor vascular targeting. This, in turn, results in a reduction of oxygen and nutrient supply within the tumor, ultimately triggering extensive tumor necrosis (Liu et al., 2022). However, it has been reported that C-NPs may lead to an increase in tumor-associated M2 macrophages (M2-TAMs) and subsequent tumor recurrence (Qin et al., 2019). To address this concern, BLZ945, a small molecule inhibitor of colony-stimulating factor-1 receptor (CSF-1R), has been introduced to induce apoptosis in M2-type macrophages within tumors (Xie et al., 2022). This helps mitigate the issue of increasing M2-TAMs caused by C-NPs. In light of this, Wang Yue et al. developed a nanomedicine by combining esterifying CA4 and BLZ945 together on PLG-g-mPEG, resulting in polymer-bonded vascular blocker and BLZ945 nanomedicines (CB-NPs). Subsequently, they conducted tumor inhibition experiments on a mouse C26 model (Wang et al., 2021). The results showed that CB-NPs had a significant inhibitory effect on the growth of tumors with large initial volumes. Huang Yue et al. further elucidated the drug loading and drug ratio of CA4 and BLZ945 in CB-NPs, highlighting their critical impact on the anti-tumor effect (Huang et al., 2023). They clarified that when the drug loading of CA4 and BLZ945 was 20.7% and the drug ratio was 0.45/1, CB-NPs showed an excellent anti-tumor effect on the H22 liver cancer model in mice and achieved a partial cure.

In order to broaden the potential applications of CB-NPs and deepen our understanding of underlying mechanisms of action, we initiated our investigation by selecting Renca renal carcinoma cell lines for *in vitro* experiments. Through CCK-8 and Transwell experiments, we confirmed that CB-NPs can effectively curb the proliferation, migration, and invasion of Renca cells. These findings, at the cellular level, attest to CB-NPs’ capability to inhibit tumor growth. To further verify the inhibitory effect of CB-NPs on tumors *in vivo*, we established a Renca renal carcinoma subcutaneous tumor bearing mouse model. Our observations, coupled with H&E staining, unequivocally demonstrated that CB-NPs had the effect of promoting tumor necrosis and inhibiting tumor growth, resulting in an impressive tumor inhibition rate of 88.0%. Building upon the validation of CB-NPs’ inhibitory effects on Renca cell lines *in vivo* and *in vitro*, we delved into unraveling the mechanism underlying CB-NPs’ tumor growth inhibition. Western blot analysis showed that CB-NPs significantly downregulated VEGFA expression, signifying their influence in inhibiting angiogenesis. Finally, we employed flow cytometry to detect the apoptotic effect of CB-NPs on Renca renal carcinoma cell lines and tumor tissue, as well as the effect on the expression of macrophages in tumor tissues. The results showed that CB-NPs exhibited a notable pro-apoptotic effect on cell apoptosis in Renca renal carcinoma cell lines, while not significantly affecting macrophages in tumor tissue, thus diminishing the risk of tumor recurrence. The above *in vivo* and *in vitro* data confirmed that CB-NPs exhibit remarkable anti-tumor effects in Renca-bearing mice, thereby introducing a novel treatment strategy for renal carcinoma.

## 2 Materials and methods

### 2.1 Materials

γ-Benzyl-L-glutamate-N-carboxyanhydride (BLG-NCA) was purchased from Chengdu enlai biological technology Co., Ltd., China. Methoxy poly (ethylene glycol) with Mw 5,000 Da (mPEG5K) was obtained from J&K Scientific Co., Ltd. The mPEGs were dried by azeotropic distillation in toluene before use. N, N′-dimethylformamide (DMF) was stored over CaH_2_ for 3 days and distilled under a vacuum prior to use. Combretastatin A4 (CA4) was purchased from Hangzhou Great Forest Biomedical Ltd., China. BLZ945 was obtained from Shanghai Bixi Chemical Co., Ltd., Shanghai. 4-Dimethylaminopyridine (DMAP) was supplied by Aladdin Reagent Co. Ltd., China. 2, 4, 6-Trichlorobenzoyl chloride was obtained from Tianjin Heowns Biochemical Technology Co., Ltd., China. All other reagents and solvents were purchased from Sinopharm Chemical Reagent Co., Ltd, China and used as received. CB-NPs were synthesized following our previous work (Huang et al., 2023). Supplementary Figures S1–S4 showed the detail characterization of CB-NPs.

### 2.2 Cell lines and animal models

The mouse renal carcinoma (Renca) cell lines were purchased from Procell under the conditions of RPMI-1640 (Seven) + 10% FBS (ABCELL) + 1% P/S (Seven) and incubated at 37°C and 5% CO_2_. Female BALB/c mice (6–8 weeks old) with an average body weight of 16–18 g were obtained from Beijing Vital River Laboratory Animal Technology Co., Ltd. All animals received proper care in compliance with the guidelines outlined in the guide for the Care and Use of Laboratory Animals and all procedures were approved by the Animal Care and Use Committee of Changchun Institute of Applied Chemistry, Chinese Academy of Sciences.

### 2.3 CCK-8 cytotoxicity experiment

The cytotoxicity of CB-NPs on Renca cells was evaluated using the CCK-8 assay. Specifically, Renca cells with a density of 4 × 10^4^ cells/mL were inoculated into a 96-well plate and cultured for 24 h before grouping. The cells were then categorized into three distinct groups: 24 h group, 48 h group, and 72 h group (*n* = 3). Within each group, various concentrations of CB-NPs were administered, comprising 0 μM, 10 μM, 50 μM, 100 μM, 200 μM, and 500 μM. Each concentration group has 3 replicate wells. After incubation for 24 h, 48 h, and 72 h, 10 μL of CCK8 were added to each well. The 96-well plate was returned to the incubator for further incubation, and after 3 h, the optical density (OD) was measured at a wavelength of 450 nm. Cell viability was calculated based on the ratio of the OD value of the sample to that of the control well.

### 2.4 Transwell cell invasion and migration experiment

Two groups, i.e., PBS group and 100 μM CB-NPs group were set for both the Transwell invasion and migration experiments. In the Transwell invasion experiment, Renca cells with a density of 2 × 10^5^ cells/mL were suspended in 600 μL of complete medium and placed in the upper chamber of a Transwell plate. The upper chamber was pre-coated with a 50 μL diluent of matrix adhesive (prepared at a ratio of 1:5 with basic culture medium) provided by Shanghai Yisheng Biotechnology Co., Ltd. Subsequently, the Transwell plate was placed within an anoxic device (Billups Rothenberg Inc., United States) to create an environment consisting of 94% nitrogen, 5% carbon dioxide, and 1% oxygen. It should be noted that all subsequent cell experiments were conducted under this anoxic condition. After incubation, the cells were fixed with methanol and stained with a rapid Giemsa staining solution (Shanghai Yisheng Biotechnology Co., Ltd.). The migration assay followed a similar protocol to the invasion assay, except that there was no matrix adhesive coating in the upper chamber. Cells that had migrated or invaded were then collected from three different fields of view using an optical microscope (Tianjin Microinstrument Optical Instrument Co., Ltd.) for statistical analysis.

### 2.5 Evaluation of subcutaneous tumor burden

Rena renal cancer cells were subcutaneously injected into female Balb/c mice (6–8 weeks old, 20 ± 2 g) at a concentration of 1.09 × 10^8^/mL, with each mouse receiving 100 μL. When the tumor volume reached −220 mm^3^, mice were randomly divided into four groups: the PBS group (Group 1), the low-dose CB-NPs group (Group 2, administrated at 20 mg/kg based on CA4 concentration; the same below), the medium-dose CB-NPs group at 30 mg/kg (Group 3) and the high-dose CB-NPs at 40 mg/kg group (Group 4). The commencement of treatment is designated as Day 0. CB-NPs were administered via tail vein injection on Day 0, Day 5, and Day 10, respectively. Tumor volume and body weight were recorded every 2 days. Tumor volume (V) was calculated using the following formula:

$$V=a\times b^{2}/2$$

Where *a* represents the longest axis and *b* represents the shortest axis of the tumor. The tumor growth inhibition rate (IR) was calculated using the following formula:

$$\text{IR}\left(\%\right)=\left(1‐\left({\text{TV}}_{t}‐{\text{TV}}_{t0}\right)/\left({\text{TV}}_{c}‐{\text{TV}}_{c0}\right)\right)\times 100$$

Where *TV*_*t*_ and *TV*_*t0*_ represent the average tumor volume at the end and beginning of the treatment group, respectively, while *TV*_*c*_ and *TV*_*c0*_ represent the average tumor volume at the end and beginning of the control group, respectively. Due to the average tumor volume of the PBS control group approaching 2,000 mm^3^ on Day 14, the experiment was recorded up to Day 14.

### 2.6 Staining with hematoxylin and eosin

For subsequent *in vivo* experiments, we selected a drug concentration of 30 mg/kg CB-NPs. Renca cells at 1.09 × 10^8^/mL, were subcutaneously injected into female Balb/c mice (6–8 weeks old, weighing 20 ± 2 g) at a volume of 100 μL per mouse. When the tumor volume reached −130 mm^3^, mice were randomly divided into two groups, i.e., the PBS group (Group 1) and the 30 mg/kg CB-NPs group (Group 2). The commencement of treatment is designated as Day 0. CB-NPs were administered via tail vein injection on Day 0 and Day 5, respectively. On Day 8, fresh tumor tissue (*n* = 3) was rinsed in PBS, fixed in 4% paraformaldehyde, embedded in wax, stained with hematoxylin and eosin (H&E), and subsequently imaged using an optical microscope (OLYMPUS) for statistical analysis.

### 2.7 qPCR in Renca cell lines and tumor tissues detection of HIF-1α, VEGFA expression

We assessed the expression of HIF-1α and VEGFA in both Renca cell lines and tumor tissues using quantitative PCR (qPCR). The experimental groups were categorized into two main sections: the PBS group and the 100 μM CB-NPs group for cell line experiments, and the PBS group and the 30 mg/kg CB-NPs grou for animal experiments. Initially, we employed the 6-min high-purity RNA extraction kit (ZS-M11005) to separate total RNA from Renca cell lines or tumor tissues. Subsequently, we conducted quantification of HIF-1α and VEGFA following reversing transcription using the Supersmart TM 6-min heat-resistant first strand cDNA synthesis kit (ZS-M14003). The primers utilized in this study were as shown in Table 1. Finally, we employed the 2^−ΔΔCT^ method for relative quantification of gene expression levels.

**TABLE 1:** The kinds and sequence of primers.

Primers	Sequence
**Mus-HIF-1α-F1**	**ATC​AGT​TGC​CAC​TTC​CCC​AC**
**Mus-HIF-1α-R1**	**TTA​ACC​CCA​TGT​ATT​TGT​TCA​CG**
**Mus-Vegfa-F1**	**CTA​CTG​CCG​TCC​GAT​TGA​GA**
**Mus-Vegfa-R1**	**TGC​TGG​CTT​TGG​TGA​GGT​TT**
**Mus-ACTIN- F1**	**CTT​TGC​AGC​TCC​TTC​GTT​GC**
**Mus-ACTIN- R1**	**CCT​TCT​GAC​CCA​TTC​CCA​CC**

### 2.8 Western blot in Renca cell lines and tumor tissues detection of HIF-1α, VEGF expression

We assessed the expression of HIF-1α and VEGFA in Renca cell lines and tumor tissues through Western blot analysis. The experimental groups were divided into two categories: the PBS group and the 100 μM CB-NPs group for cell experiments, and the PBS group and the 30 mg/kg CB-NPs group for animal experiments. To begin with, we utilized the Minute™ Total Protein Extraction Kit (Invent) to extract total proteins from Renca cell lines or tumor tissues and measure the concentration of the extracted proteins using the BCA method. Subsequently, the proteins were separated by polyacrylamide gel and transferred onto a PVDF membrane, which was sealed with TBS-T solution for 2 h. The membrane was then incubated with the primary antibody, including β-Actin (RA1012), VEGFA (19003-1-AP), HIF1α (340462). Following the incubation, the membrane was exposed to secondary antibodies at room temperature for 1.5 h. The protein bands were visualized using a chemiluminescence imaging system (Clinx, China). The molecular weight relied on the identity of the proteins based on antibody staining.

### 2.9 Immunohistochemical staining

The expression of HIF-1α and VEGFA in tumor tissues of Renca-bearing mice was analyzed using immunohistochemical staining. The experiment was divided into the PBS group and the 30 mg/kg CB-NPs group. The initial steps of the experiment were consistent with the preparation for H&E staining. Tumor tissues (*n* = 3) were collected and rinsed in PBS, fixed in 4% paraformaldehyde, embedded in wax, and sectioned. The sections were then subjected to staining using HIF-1α Antibodies (ZENBIO, 340462) and VEGFA antibodies (protenteh, 19003-1-AP). Images were obtained using an optical microscope (OLYMPUS) for subsequent statistical analysis.

### 2.10 Apoptosis assay

We assessed cell apoptosis through flow cytometry (FCM) analysis. The experimental groups were divided into two sections: the PBS group and the 100 μM CB-NPs group for cell experiments, and the PBS group and the 30 mg/kg CB-NPs group for animal experiments. For Renca cells, they were prepared either as suspension cells or tumor tissue was cut and digested to create a cell suspension (*n* = 3). These cell suspensions were then stained with Annexin V-FITC (Biogene) and subjected to flow cytometry analysis using a BD Bioscience flow cytometer.

### 2.11 Changes in M1 and M2 expression in macrophages

We analyzed the changes in macrophages expression through flow cytometry (FCM). The experiment groups were divided into two sections: the PBS group and the 30 mg/kg CB-NPs group. Tumor tissue was cut and digested to prepare a cell suspension, and the cell count was adjusted to 1 × 10^6^/100 μL. For each group (PBS and CB-NPs, *n* = 3), 100 μL of the cell suspension was taken, and 2 μL of CD16/32 was added while keeping the samples on ice. Corresponding volumes of fluorescent antibodies were added, which included PerCP anti-mouse F4/80 (123125, Biolegend), APC anti-mouse/human CD11b (101211, Biolegend), APC/Cyanine7 anti-mouse CD86 (105029, Biolegend), and PE/Cyanine7 anti-mouse CD206 (MMR) (141719, Biolegend). The samples were then incubated at room temperature, shielded from light, for 30 min. After incubation, the supernatant was removed by centrifugation, and the cells were resuspended in 200 μL of CSB. The stained cells were analyzed using a flow cytometer (EXFLOW-206, Dakota).

### 2.12 Statistic analysis

Data are expressed as mean ± standard deviation (SD). Statistical differences between groups were analyzed using one-way analysis of variance (ANOVA). Group comparisons were further evaluated with t-tests. Significance levels were defined as follows: *p < 0.05 (considered statistically significant), **p < 0.01 (highly significant), ***p < 0.001 (extremely significant), and ****p < 0.0001 (incredibly significant), “ns” indicates that there was no statistically significant difference.

## 3 Results and discussion

Formula optimization and *in vivo* study of poly (L-glutamic acid)-*g*-methoxy poly (ethylene glycol)/combretastatin A4/BLZ945 nanoparticles for cancer therapy has been completed as described in one of our previous studies (Huang et al., 2023). Unlike cytotoxic drugs, which only exhibit their killing effect when they come into contact with tumor cells (Weaver, 2014), VDAs induce extensive necrosis within tumors by disrupting the tumor vasculature required for tumor development and progression (Al-Abd et al., 2017; Ho et al., 2017; Johnson et al., 2019; Cermak et al., 2020), thereby inhibiting tumor growth. As previously mentioned (Wang et al., 2021), CB-NPs are polymeric nanodrugs co-formulated with CA4 and BLZ945, demonstrating their ability to inhibit tumor cell growth in various tumor models. In this study, we not only expanded the scope of its applicability but also verified its anti-tumor efficacy in the Renca renal cancer model, shedding light on its underlying mechanism.

### 3.1 The effect of CB-NPs on the Renca renal cell carcinoma cell line

**FIGURE 1:**
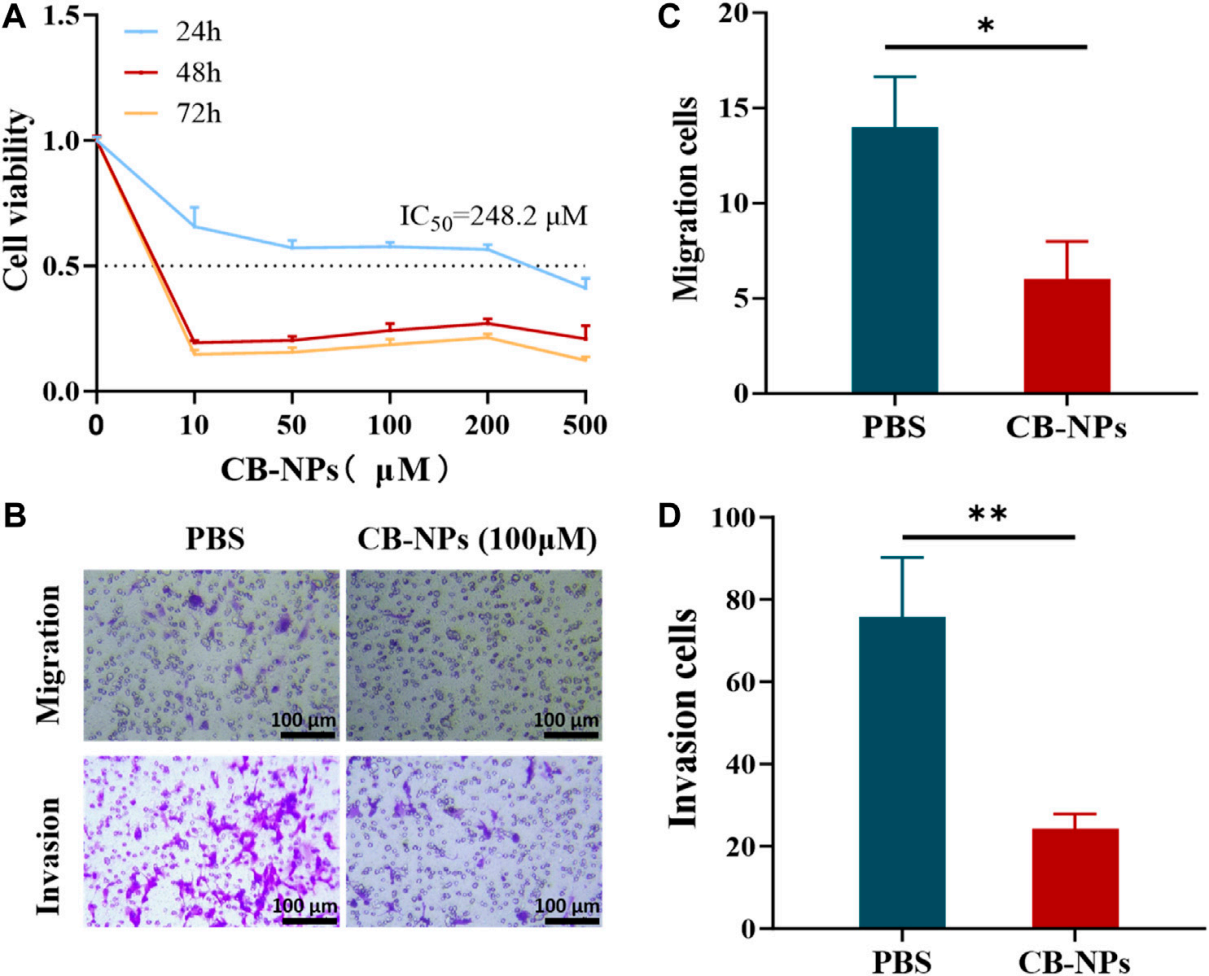
Impacts of CB-NPs on the Renca renal carcinoma cell line. **(A)** The cytotoxicity of CB-NPs on the Renca cell line was detected using the CCK-8 method (*n* = 3). **(B)** The Transwell cell migration and invasion experiments were performed on the Renca cell line, and the results were observed under a ×200 magnification (*n* = 3). **(C)** Significant difference observed in Transwell cell migration between PBS and CB-NPs groups, *p < 0.05. **(D)** Significant difference observed in Transwell cell invasion between PBS and CB-NPs groups, **p < 0.01.

We assessed the cytotoxicity of CB-NPs on the Renca cell line using the CCK-8 assay (Figure 1A). The results of the 24-h CCK-8 cytotoxicity test showed that the toxicity of CB-NPs to the Renca cell line was drug concentration-dependent, with the half maximal inhibitory concentration (IC_50_) determined to be 248.2 μM through nonlinear fitting. Subsequent 48-h and 72-h CCK-8 cytotoxicity tests demonstrated a potent cell-killing effect at a concentration of 10 μM CB-NPs. As the concentration increased, the activity of the Renca cell line tended stabilize. Therefore, in subsequent cell experiments, 100 μM was selected and the cells were subjected to hypoxia treatment for 48 h to simulate the hypoxic environment on tumors.

To investigate the effects of CB-NPs on the migration and invasion of the Renca cell line, we conducted the Transwell experiment (Figure 1B). Upon observation and counting under a 200 × microscope for statistical analysis, it became evident that the CB-NPs group significantly suppressed both cell migration and invasion of the Renca cell line compared to the PBS group (Figures 1C, D).

The above cell experiment results indicate that CB-NPs exhibit cytotoxicity towards the Renca cell line, effectively inhibiting their migration, proliferation, and ultimately leading to a reduction in tumor growth at the cellular level.

### 3.2 The effect of CB-NPs on Renca tumor-bearing mice

**FIGURE 2:**
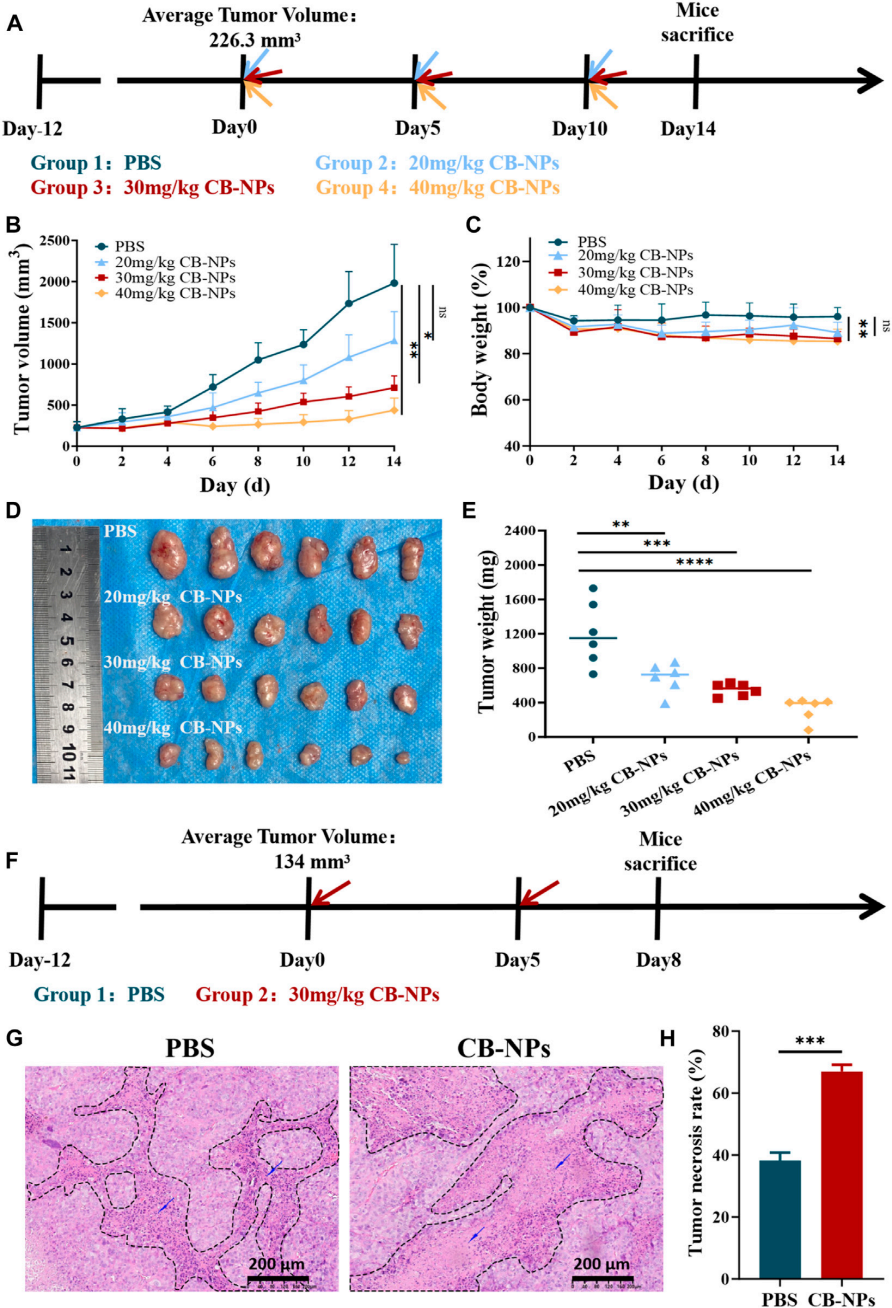
Impacts of CB-NPs on mice with renal cancer. **(A)** Timeline depicting the drug administration for tumor inhibition experiments (*n* = 6). **(B)** Growth curve of subcutaneous Renca renal cancer tumors, with significance levels indicated as follows: ns *p* > 0.05, *p < 0.05, **p < 0.01. **(C)** Body weight change curve of Renca-bearing mice, with significance levels indicated as follows: ns *p* > 0.05, **p < 0.01. **(D)** Images of the physical appearance of subcutaneous Renca renal cancer tumors. **(E)** Mass of subcutaneous Renca renal cancer tumors, with significance levels indicated as follows: **p < 0.01, ***p < 0.001, ****p < 0.0001. **(F)** Timeline illustrating H&E administration (*n* = 3). **(G)** Microscopic images of H&E staining, scale bar 200 μm. **(H)** Analysis of H&E staining results, with significance indicated as follows: ***p < 0.001.

We further evaluated the anti-tumor efficacy of CB-NPs *in vivo* using Renca tumor-bearing mice. When the average tumor volume of the mice reached −220 mm^3^, they were randomly divided into 4 groups (*n* = 6). The initiation of treatment was designated as Day 0. On Days 0, 5, and 10, the following different treatment plans were administered through the caudal vein: PBS, 20 mg/kg CB-NPs (based on CA4 concentration; the same below), 30 mg/kg CB-NPs and 40 mg/kg CB-NPs. The results were monitored until the end of Day 14 (Figure 2A). Statistical analysis indicated that the effect of CB-NPs on tumors exhibited a dose-dependent relationship. Higher dose of CB-NPs led to more pronounced tumor inhibition (Figure 2B). On the Day 14, the tumor suppression rate (TSR, %) was calculated and it increased from low to high, measuring 39.7%, 72.4%, and 88.0% (Figure 2D). Additionally, tumor mass decreased with the increased CB-NPs dosage (Figure 2E), indicating that CB-NPs can reduce tumor size in Renca tumor-bearing mice. However, it is worthy noting that the body weight of mice decreased with the increasing dosage (Figure 2C), suggesting that CB-NPs have a certain toxic effect on the Renca tumor-bearing mouse model.

To verify the effect of CB-NPs on the morphology of tumor tissue in Renca tumor-bearing mice, 30 mg/kg CB-NPs was used as the dose concentration to avoid higher toxicity of CB-NPs at a dosage above this level. When the tumor volume reached −130 mm^3^, the mice were randomly divided into two groups: the PBS group and the 30 mg/kg CB-NPs group. The commencement of treatment was designated as Day 0, and CB-NPs were administrated via the tail vein on Day 0 and Day 5, respectively. The mice were euthanized on Day 8 (Figure 2F) and the tumor samples were collected for H&E staining. Microscopic examination revealed a substantial presence of nuclear Under the microscope, a large number of fragments of nuclear lysis fragments in the necrotic area, with a higher degree of nuclear fragmentation observed in the CB-NPs group compared to the PBS group (Figure 2G). Further statistical results confirmed a significantly greater extent of necrosis in the tumors following CB-NPs administration compared to the PBS group (Figure 2H).

The above *in vivo* experiments provide evidence that CB-NPs effectively inhibit tumor growth mice in Renca tumor-bearing mice. This inhibitory effect is positively correlated with the concentration of CB-NPs. Meanwhile, CB-NPs can cause extensive necrosis within tumors, further demonstrating the inhibitory effect on tumor growth.

### 3.3 The effect of CB-NPs on tumor angiogenesis

As the tumor grows, tumor cells and other cells in the tumor microenvironment (TME) gradually become hypoxic, triggering the activation of hypoxia signaling (Yuan et al., 2023). This condition leads to vascular abnormalities, inadequate blood supply, and, ultimately, the development of more aggressive and drug-resistant tumors (Kopecka et al., 2021; Tao et al., 2021). Hypoxia-inducible factor (HIF) plays a pivotal role in various aspects of tumor development, including angiogenesis, tumor cell proliferation, and metastasis (Kaelin and Ratcliffe, 2008; Baldewijns et al., 2010), HIF-1α is one of the subunits of HIF, and there are many opinions about its role in tumors. HIF-1α, one of HIF’s subunits, has been the subject of different interpretations regarding its acts as a tumor suppressor gene (Gonzalez et al., 2018; Mazumder et al., 2023). On the other hand, there is evidence that HIF-1α directly or indirectly regulates genes related to tumor proliferation, tumor cell apoptosis, metastasis, and invasion (Rankin and Giaccia, 2016; Rust et al., 2019), and even the expression of angiogenic factors including vascular endothelial growth factor (VEGF) (Wilson and Hay, 2011). VEGF is a key factor involved in tumor angiogenesis and promotes tumor progression (Fukumoto et al., 2023).

In our prior experiments, we observed that CB-NPs can lead to tumor necrosis. This outcome is theoretically attributed to the ability of CB-NPs to block tumor blood vessels, resulting in reduced nutrient and blood supply within the tumor. We hypothesize a close connection between this effect and hypoxia and angiogenesis. To confirm this hypothesis, we conducted assessments of HIF-1α and VEGFA expression at both the gene and protein levels through qPCR and Western blot analyses. Subsequently, we further examined the expression of HIF-1α and VEGFA in tumor tissues using immunohistochemistry.

**FIGURE 3:**
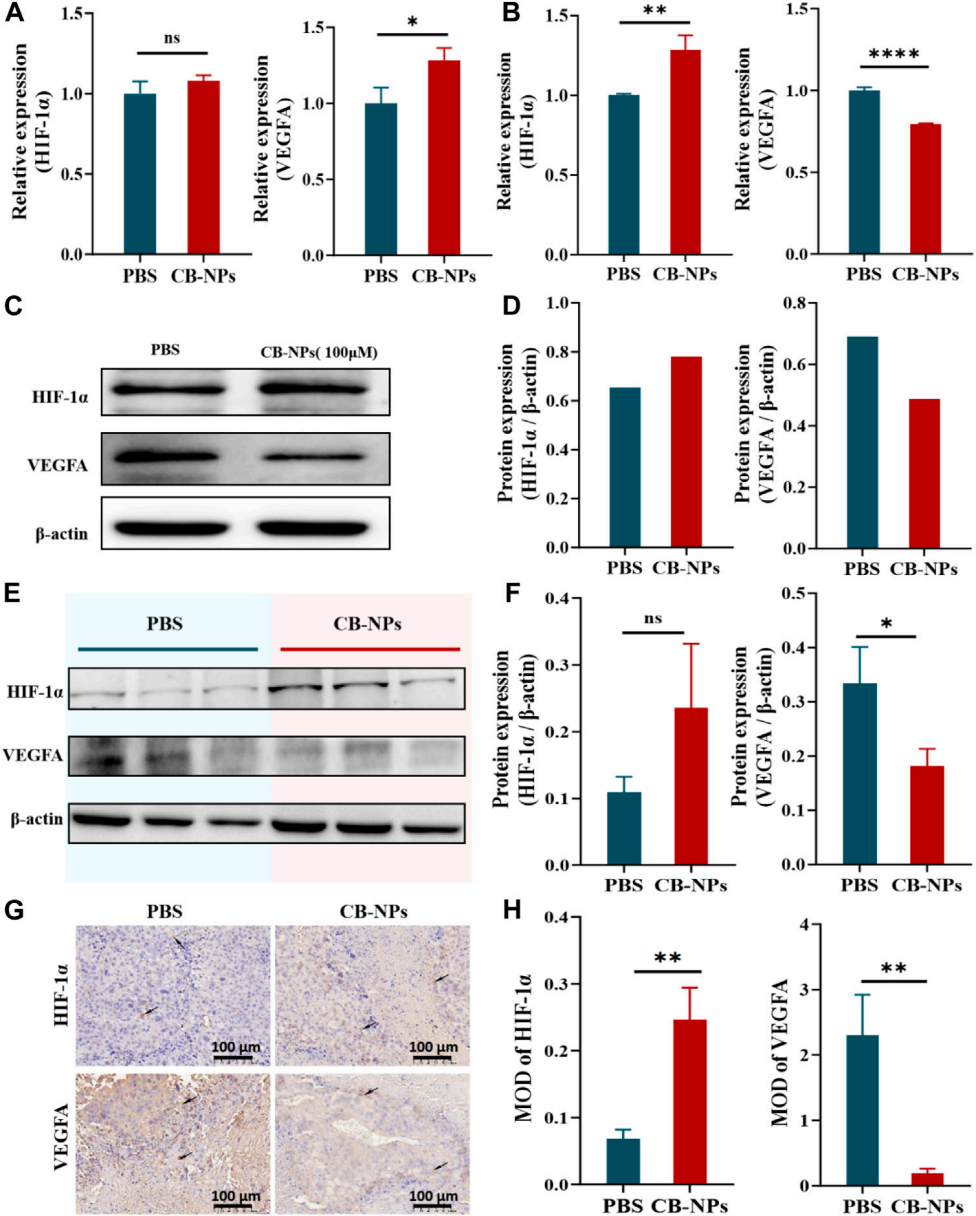
Impacts of CB-NPs on tumor angiogenesis. **(A)** qPCR experiment in the Renca cell line (*n* = 3), ns *p* > 0.05, *p < 0.05. **(B)** qPCR experiment in the tumor tissue of Renca-bearing mice (*n* = 3), **p < 0.01, ****p < 0.0001. **(C)** Western blot experiment in the Renca cell line. **(D)** Grayscale band analysis of Western blot in the Renca cell line. **(E)** Western blot experiment in the tumor tissue of Renca-bearing mice (*n* = 3). **(F)** Grayscale band analysis of Western blot in the tumor tissue of Renca-bearing mice, ns *p* > 0.05, *p < 0.05. **(G)** Immunohistochemical staining under the microscope, scale bar 100 μm (*n* = 3). **(H)** Immunohistochemical analysis, **p < 0.01.

We first employed qPCR to detect the expression changes of HIF-1α and VEGFA in the Renca cell line. Compared to the PBS group, the expression of HIF-1α in the CB-NPs group was slightly higher despite being insignificant. By contrast, the expression of VEGFA exhibited a significant increase (Figure 3A). Subsequently, we conducted Western blot analysis to provide further insights into the expression of HIF-1α and VEGFA in the Renca cell line. The results revealed an elevation in HIF-1α expression in the CB-NPs group, while VEGFA expression decreased (Figure 3C). This expression trend is more clearly illustrated in the grayscale band analysis (Figure 3D). The discordant expression patterns of VEGFA observed in qPCR and Western blot in the cell experiment may be due to processing and degradation during gene transcription and translation, leading to incomplete consistency between transcription and translation levels.

We proceeded to confirm the expression of HIF-1α and VEGFA in tumor tissues of Renca-bearing mice. In the qPCR results, it was evident that the CB-NPs group exhibited a significant increase in HIF-1α expression compared to the PBS group (Figure 3B), while VEGFA expression significantly decreased compared to the PBS group (Figure 3B). In the Western blot results, the CB-NPs group displayed elevated HIF-1α expression and reduced VEGFA expression relative to the PBS group (Figure 3E). Greyscale band analysis revealed no significant difference in the increase in HIF-1α expression (Figure 3F), while a marked difference was observed in the decrease in VEGFA expression (Figure 3F).

Finally, we also explored the expression of HIF-1α and VEGFA in tumor tissues of Renca-bearing mice by immunohistochemical staining (Figure 3G). The results were quantified by mean optical density (MOD), and showed that CB-NPs significantly increased the expression of HIF-1α and significantly decreased the expression of VEGFA in tumor tissues (Figure 3H).

In light of the substantial decrease in VEGFA expression, we speculate that CB-NPs might inhibit tumor angiogenesis to some extent, resulting in a reduction in oxygen and nutrient supply within the tumor. This, in turn, triggers necrosis within the tumor and elevates the expression of HIF-1α, ultimately inhibiting tumor growth. In essence, the increase in HIF-1α appears to be associated with the decrease in VEGFA expression. This conclusion aligns with the results reported by Ryo Onodera (Onodera et al., 2023).

### 3.4 The effect of CB-NPs on tumor cell apoptosis and macrophage count

Tumor-associated macrophages (TAMs) are tumor-infiltrating immune cells that can be differentiated into M1 and M2 phenotypes, usually influenced by the TME (Mantovani and Locati, 2013). Among them, M1 is defined as a classically activated macrophage that can activate the immune response, phagocytize and kill cancer cells, and inhibit their activity. M2 is defined as a surrogate-activated macrophage that promotes tumor invasion and metastasis, angiogenesis, and cancer cell activity (Yang et al., 2023). CSF1R-mediated signaling is crucial for the differentiation and survival of macrophages, which can effectively prevent tumor metastasis (Stanley and Chitu, 2014; Cannarile et al., 2017; Rodriguez-Tirado et al., 2022). BLZ945 is a highly selective small-molecule CSF-1R inhibitor that depletes TAMs by blocking CSF-1R, leading to a reduction in M2-TAM infiltration (Cannarile et al., 2017; Xie et al., 2022). To determine whether BLZ945 plays a role in CB-NPs, further investigation through flow cytometry is needed. This will help assess the effects of CB-NPs, especially in terms of macrophage polarization and the impact of BLZ945 on these processes.

**FIGURE 4:**
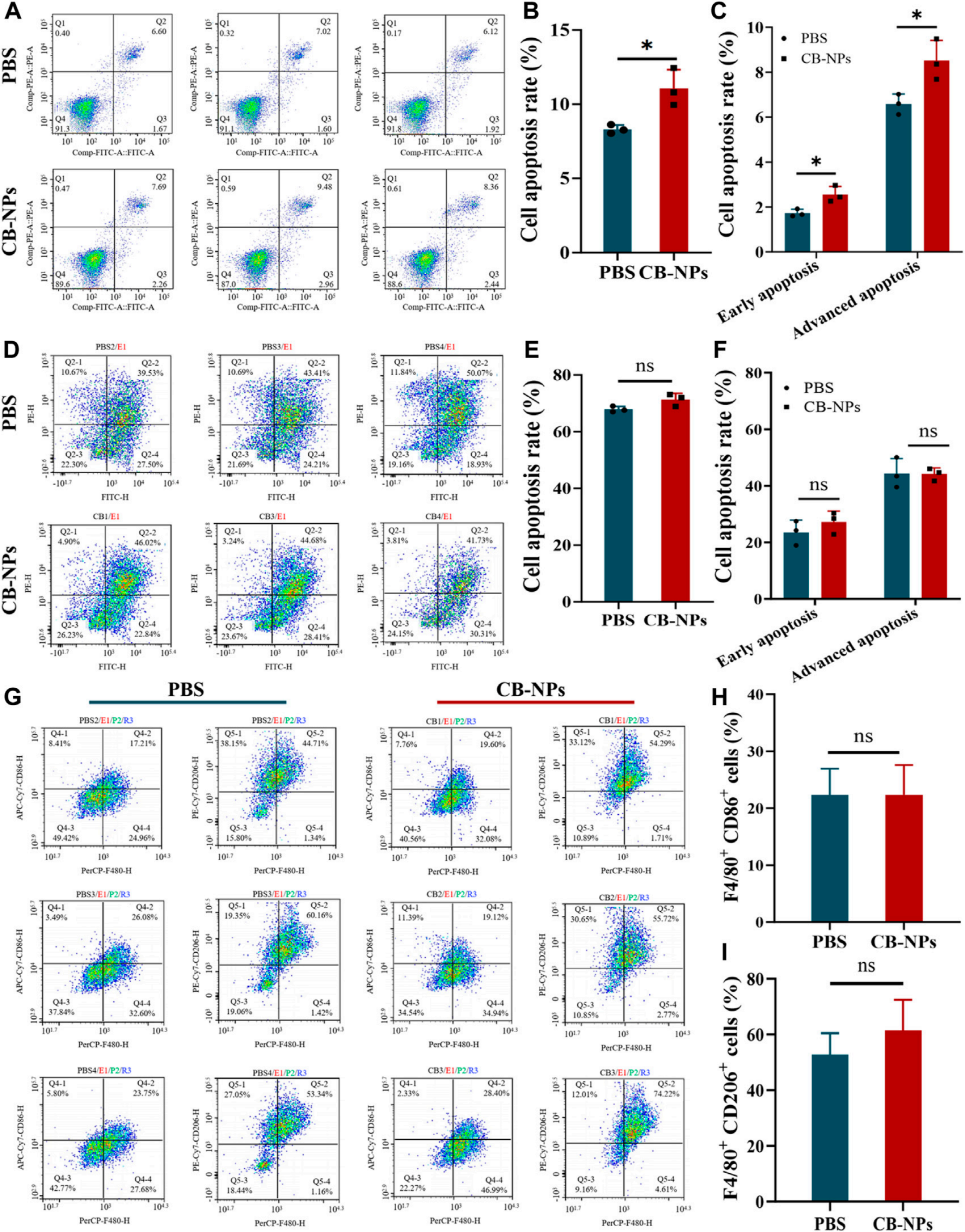
Effects of CB-NPs on the number of apoptotic tumor cells and macrophages. **(A)** Flow cytometry apoptosis detection of Renca cell line (*n* = 3). **(B)** Renca cell lines apoptosis ratio diagram, *p < 0.05. **(C)** Renca cell lines early and late apoptosis ratio diagram, *p < 0.05. **(D)** Flow cytometry apoptosis detection of tumor tissue in Renca tumor-bearing mice (*n* = 3). **(E)** Renca tumor-bearing mice tumor tissue apoptosis ratio diagram, ns *p* > 0.05. **(F)** Renca tumor-bearing mice tumor tissue early and late apoptosis ratio diagram, ns *p* > 0.05. **(G)** Flow cytometry detection of M1 and M2 macrophage number changes in tumor tissue of Renca tumor-bearing mice (*n* = 3). **(H)** M1 macrophage proportion diagram, ns *p* > 0.05. **(I)** M2 macrophage proportion diagram, ns *p* > 0.05.

The Annexin V-FITC double staining method was used to determine the effect of CB-NPs on tumor apoptosis in the Renca cell line and Renca tumor-bearing mice. The results in Figures 4A,D depict the flow cytometry findings for the Renca cell line and tumor cells of Renca tumor-bearing mice. The Q2 region shows late apoptotic cells, while the Q3 region shows early apoptotic cells. The total apoptotic cell ratio is the sum of the late apoptotic cell ratio and early apoptotic cell ratio. The subsequent statistical analysis revealed that CB-NPs significantly increased the proportion of total apoptosis, early apoptosis, and late apoptosis in the Renca cell line (Figures 4B, C). However, CB-NPs had no significant effect on the various apoptosis stages of Renca tumor-bearing mice (Figures 4E, F). From a cellular perspective, the use of CB-NPs led to a significant increase in the proportion of cell apoptosis in each stage, indicating that CB-NPs have a certain pro-apoptotic effect on Renca cell line. However, there was no significant difference in the effect on tumor tissue, which might be due to the complex microenvironment within the tumor.

To investigate the effect of CB-NPs on the number of macrophages in Renca tumor-bearing mice, flow cytometry was employed to detect the positive expression of CD86 and CD206 (Figure 4G). The Q4-2 region represents the number of F4/80^+^CD86^+^ cells, indicating M1 macrophages, while the Q5-2 region represents the number of F4/80^+^CD206^+^ cells, indicating M2 macrophages. Statistical analysis suggested that CB-NPs had no significant effect on the number of M1 and M2 cells in tumor tissue (Figures 4H, I). Previous studies suggested that using CA4 alone could cause a significant increase in M2 macrophages, which might lead to tumor recurrence (Qin et al., 2019). The flow cytometry findings in the CB-NPs group showed no significant increase in M2 macrophages compared to the PBS group. This may suggest that CB-NPs inhibited the polarization of M2 macrophages and prevented tumor recurrence.

In summary, our finding demonstrate that CB-NPs stimulate apoptosis in the Renca cell line, suppress the abundance of M2 macrophages within Renca tumor-bearing mice, inhibit tumor invasion and metastases, and mitigate the potential for tumor recurrence.

## 4 Conclusion

Our study confirms the remarkable therapeutic efficacy of CB-NPs on the Renca tumor-bearing mouse model, expanding the scope of the application of CB-NPs. Furthermore, it delves deeper into the mechanisms through which CB-NPs inhibit tumor growth. CB-NPs can reduce the expression of VEGFA and restrain tumor angiogenesis to impede tumor progression. It also exhibit cytotoxicity towards the Renca cell line, effectively inhibiting its migration, proliferation, and ultimately leading to a reduction in tumor growth at the cellular level. By using flow cytometry, we have determined that there is no significant difference in the effect of CB-NPs on the quantity of M1 and M2 macrophages in Renca tumor-bearing mice. Based on previous data on C-NPs treatment of tumors, a substantial increase in the number of M2 macrophages in the later stages (Qin et al., 2019). Our study indicates that CB-NPs inhibit the significant increase of M2 macrophages in Renca tumor-bearing mice, which could reduce the possibility of tumor recurrence. These findings underscore the potential of CB-NPs to effectively inhibit the progression of renal carcinoma in the Renca-tumor bearing mouse model, offering a novel therapeutic approach for the treatment of renal carcinoma.

## Data Availability

The datasets presented in this study can be found in online repositories. The names of the repository/repositories and accession number(s) can be found in the article/Supplementary Material.
