# Supplementary material for: Therapeutic effects and underlying mechanism of poly (L-glutamic acid)-g-methoxy poly (ethylene glycol)/combretastatin A4/BLZ945 nanoparticles on Renca renal carcinoma
Source: Front Bioeng Biotechnol. 2024 Feb 5;12:1336692. doi: 10.3389/fbioe.2024.1336692 (PMC10875097; doi:10.3389/fbioe.2024.1336692)
Supplement: Supplementary file 1 [file DataSheet1.doc]

***Supplementary Material***

**Therapeutic Effects and Underlying Mechanism of Poly(L-glutamic acid)-*g*-methoxy poly(ethylene glycol)/combretastatin A4/BLZ945 Nanoparticles on Renca Renal Carcinoma**

1. **Supplementary Data**

**Scheme S1.** Synthetic route of CB-NPs.

**Figure S1.** ^1^H NMR of CB-NPs.

**Figure S2.** DLS of CB-NPs.

**Figure S3.** GPS pattern of CB-NPs.

**Figure S4.** HPLC pattern of CB-NPs.

- 1. **Synthesis and Characterization**


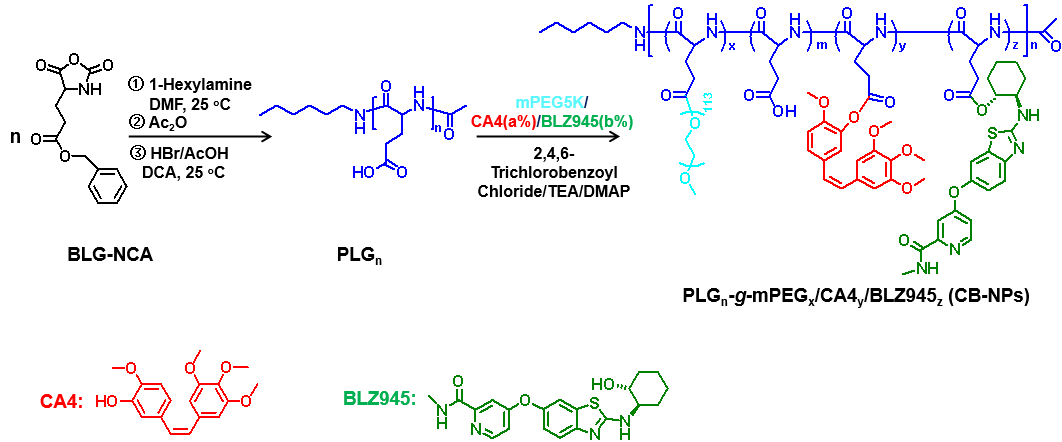


**Scheme S1.** Synthetic route of CB-NPs.

**1.2 Synthesis of poly(L-glutamic acid)s (PLG_n_)**

Poly(L-glutamic acid)s (PLG_n_) were synthesized as reported in our previous work ^1^. Briefly, γ-Benzyl-L-glutamate-N-carboxyanhydride (BLG-NCA) was dissolved in anhydrous DMF under a nitrogen atmosphere. Then n-hexylamine in anhydrous DMF was added. The polymerization was carried out at 25 ℃ for 3 days. At the end of 3 days, acetic anhydride was added to further react for 24 h at 25 ℃. Reaction mixture was precipitated into the excessive ether. A white solid poly(γ-benzyl L-glutamate) (PBLG) was obtained after drying under vacuum at room temperature for 24 h. ^1^H NMR (300M, CF_3_COOD) of PBLG: δ 7.24 ppm (br, 5H, -CH_2_C_6_***H***_5_), 5.11 ppm (br, 2H, -C***H***_2_C_6_H_5_), 4.66 ppm (br, 1H, -C***H***<), 2.48 ppm (br, 2H, -C***H***_2_COO-), 2.13 and 1.96 ppm (br, 2H, -CHC***H***_2_-). The PBLG was dissolved in dichloroacetic acid (DCA) and then HBr/acetic acid (33 wt%) was added (Weight of PBLG/Volume of DCA/Volume of HBr/acetic acid = 1 g/10 mL/4 mL). The solution was stirred at 30 ℃ for 1 h before precipitation into the excess ether. After drying under vacuum, the precipitate was dialyzed (MWCO 3500) against distilled water and freeze-dried to give the PLG product in white powders. ^1^H NMR (400M, NaOD/D_2_O) of PLG: δ 4.10 ppm (t, 1H, -C***H***<), 2.06 ppm (m, 2H, -C***H***_2_COOH), 1.82 and 1.72 ppm (m, 2H, -CHC***H_2_***-). PLG_n_ with a certain polymerization degree was produced by calculated the feed ratio of monomer to initiator. Here “n” represents the molar ratio of BLG-NCA to n-hexylamine in feed. According to our screening work, when “n” was 160, PLG performed perfect properties for the next grafting operation.

**1.3 Synthesis of poly(L-glutamic acid)-*g*-methoxy poly(ethylene glycol)/combretastatin A4/BLZ945 (****PLG_160_-*g*-mPEG_8.30_/CA4_7.50_/BLZ945_4.20_)**

PLG_160_-*g*-mPEG_8.30_/CA4_7.50_/BLZ945_4.20_ (CB-NPs) were prepared by the Yamaguchi reaction of PLG with mPEG, CA4 and BLZ945. The content determination of CA4 and BLZ945 had been discussed in our previous work^2^. Typically, PLG, dried mPEG, CA4 and BLZ945 were dissolved in anhydrous DMF by heating at 40 ℃ for 2 hours. After the temperature was cooled to 25 ℃, 2,4,6-trichlorobenzoyl chloride, triethylamine and DMAP were added in succession. After stirring at 25 ℃ for 24 h, the reaction mixture was precipitated into the excess ether and washed twice with ether. After drying under vacuum, the precipitate was dialyzed (MWCO 3500) against distilled water and freeze-dried to give the PLG_160_-*g*-mPEG_8.30_/CA4_7.50_ /BLZ945_4.20_ product as active pharmaceutical ingredients (API). ^1^H NMR (300M, D_2_O/NaOD/DMSO-*d_6_*), DLS, GPC (DMF), and HPLC spectra of CB-NPs were shown in Fig. S1-S4.

1. **Figures and Tables**


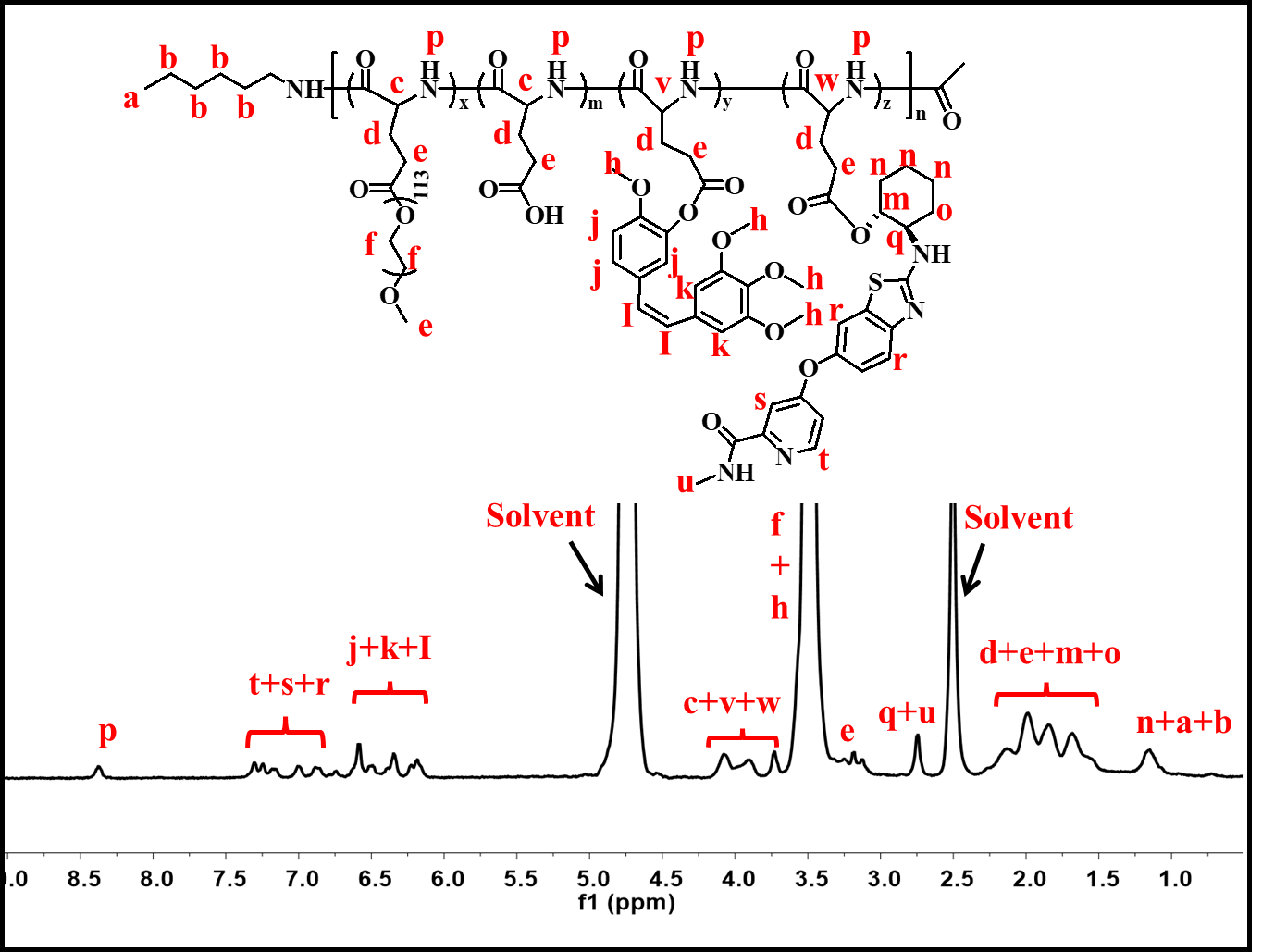


**Figure S1.** The ^1^H NMR of CB-NPs.

**
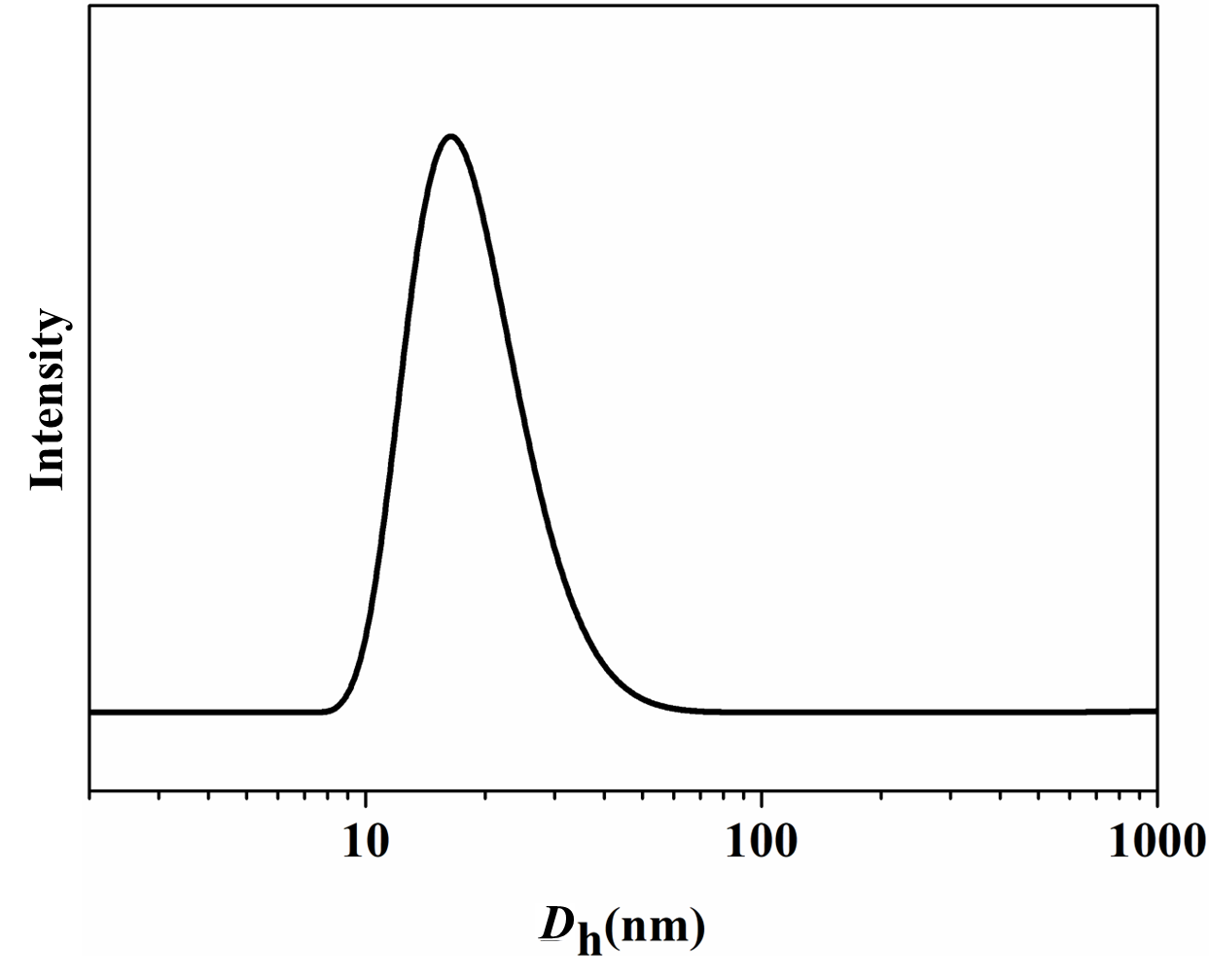
**

**Figure S2.** The DLS of CB-NPs.

**
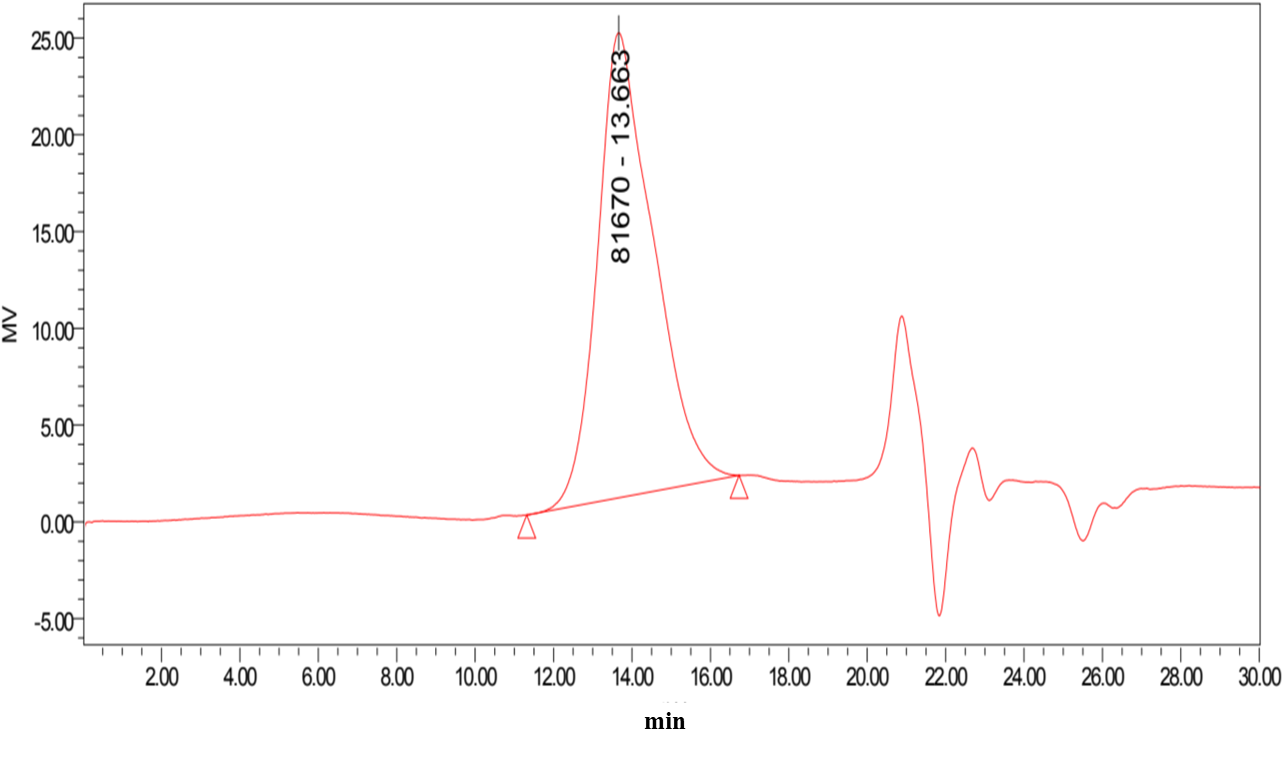
**

**Figure S3.** The GPC (DMF) pattern of CB-NPs.

**
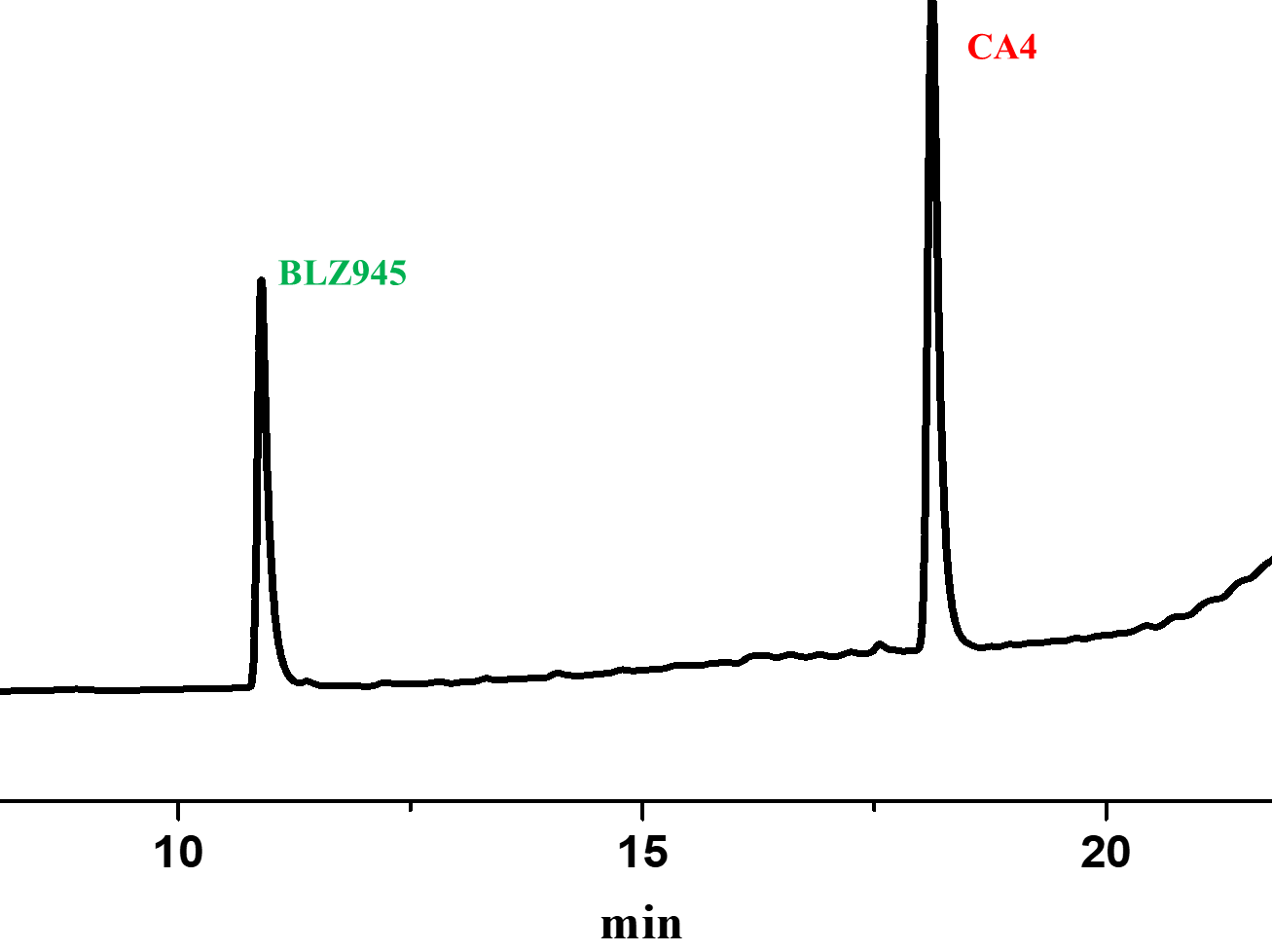
**

**Figure S4.** The HPLC pattern of CB-NPs after hydrolyzed by NaOH.

1. **Reference**

1. Wang, Y.; Shen, N.; Wang, Y.; Zhang, Y.; Tang, Z.; Chen, X., Self-Amplifying Nanotherapeutic Drugs Homing to Tumors in a Manner of Chain Reaction. *Adv Mater* **2021,** *33* (7), e2002094.

2. Huang, Y.; Yang, C.; Lv, J.; Zhang, Y.; Wang, K.; Ma, L.; Liu, Z.; Yu, H.; Li, M.; Tang, Z., Formula Optimization and In Vivo Study of Poly(L-glutamic acid)-g-methoxy poly(ethylene glycol)/combretastatin A4/BLZ945 Nanoparticles for Cancer Therapy. *Int J Pharm* **2023,** *636*, 122849.
